# Early evaluation of the transition from an analog to an electronic surgical logbook system in Sierra Leone

**DOI:** 10.1186/s12909-021-03012-z

**Published:** 2021-11-15

**Authors:** Sophia Sung, Hilde Hørthe, Øyvind Veel Svendsen, Alex J. van Duinen, Øyvind Salvesen, Alphonsus Vandi, Håkon A. Bolkan

**Affiliations:** 1 Department of Clinical and Molecular Medicine, Norwegian University of Science and Technology (NTNU), P.O. Box 8905 MTFS, 7491 Trondheim, Norway; 2 CapaCare, c/o Dr Håkon Bolkan, Clinic of Surgery, St. Olavs Hospital, P.O. Box 3250 Sluppen, 7006 Trondheim, Norway; 3 Clinic of Internal Medicine and Rehabilitation, Levanger Hospital, P.O. Box 333, 7601 Levanger, Norway; 4 Clinic of Surgery, St. Olavs Hospital, P.O. Box 3250 Sluppen, 7006 Trondheim, Norway

**Keywords:** Surgical training, Medical education, Global surgery, Mobile applications, Surgical logbook, Database, eHealth

## Abstract

**Background:**

Surgical logbooks are a commonly used tool for quality assurance of surgical training. Electronic logbooks are increasingly applied in low-resource settings, but there is limited research on their quality. The aim of this study is to evaluate the quality of an app-based surgical e-logbook system shortly after its implementation in a low-income country and to identify potential areas of improvement for the system.

**Methods:**

Entries in the e-logbook system were cross-checked with hospital records and categorized as matched or overreported. Moreover, the hospital records were checked for underreported procedures. Additionally, semi-structured interviews were conducted with users of the e-logbook system.

**Results:**

A total of 278 e-logbook database entries and 379 procedures in the hospital records from 14 users were analyzed. Matches were found in the hospital records for 67.3% of the database entries. Moreover, 32.7% of the database entries were overreported and 50.7% of the procedures in the hospital records were underreported. A previous study of an analog surgical logbook system in the same setting estimated that 73.1% of the entries were matches or close matches. Interviews with 12 e-logbook users found overall satisfaction but also identified potential areas of improvement, including the need for more training in the use of the system, modifications to improve user-friendliness, and better access to the necessary technology.

**Conclusions:**

A reliable documentation system is necessary to evaluate the quality of health workforce training. The early evaluation of a surgical e-logbook system in a low-income country showed that the collected data should be approached with caution. The quantitative analysis suggests that the e-logbook system needs to be improved in terms of accuracy. In interviews, users reported that digitalization of the logbook system was a much-needed innovation but also identified important areas of improvement. Recognition of these aspects at an early stage facilitates guidance and adjustment of further implementation and might improve the accuracy of the system.

**Supplementary Information:**

The online version contains supplementary material available at 10.1186/s12909-021-03012-z.

## Background

Less than 5% of the more than 3 billion people living in low- and lower-middle-income countries have access to timely and affordable surgical and anesthetic care should they need it [1]. A major limiting factor for accessible surgical care worldwide is the lack of human resources [2]. The Lancet Commission on Global Surgery [2] estimated in 2015 that an additional 2.28 million surgical providers are needed by 2030 to meet global surgical needs.

One measure to ensure quality training of new surgical providers is monitoring progress and development using a surgical logbook [3, 4]. Electronic (e-) logbooks are an established practice in high-income countries [3–5] and are increasingly applied in low-income countries (LICs) [6, 7]. However, there is limited documentation on the quality of surgical e-logbooks in LICs, especially in the early phase of implementation [8]. An early evaluation of eHealth solutions facilitates guidance and adjustment of further implementation and has been described as essential to increase the likelihood of successful adoption [8, 9].

The aim of this study is to evaluate the quality of an app-based surgical e-logbook system shortly after implementation in an LIC and to identify potential areas of improvement for the system.

## Methods

### The study

In this mixed-methods study, the quantitative aspect compares surgical procedures recorded in hospital records (HRs) with database entries for surgical procedures in an e-logbook system. The qualitative component entails semi-structured interviews with users of the e-logbook system.

### Setting

Sierra Leone is an LIC [10] located in West Africa that ranks 182 of 189 countries on the Human Development Index [11]. Access to surgical care is limited, with an estimated prevalence of untreated surgical conditions of 25% for the general population [12].

Since 2011, the Sierra Leonean Ministry of Health and Sanitation, the United Nations Population Fund (UNFPA), and the nonprofit organization CapaCare have jointly offered an innovative nationwide surgical training program (STP) for associate clinicians [13]. This program aims to improve access to emergency surgical and obstetric care in rural areas through the concept of task-sharing.

Since the inception of the training program a decade ago, a surgical logbook system has been used to monitor the surgical exposure and progress of trainees and graduates [14]. Initially, this system consisted of handwritten personal logbooks, from which the information was transferred to Microsoft® Excel and then emailed to a central database. An evaluation of this analog logbook system performed in 2016 by Svendsen and Helgerud et al. [14] identified inaccuracies and labor-intensive follow-up and recommended the development of a fully digital e-logbook. This recommendation was supported by a study in Uganda that reported that an electronic database captured 97.5% of the operation logbook entries [15].

Through an innovation seed fund, CapaCare has developed a surgical logbook application (app) for Android™ devices in collaboration with an independent app developer, and the company CheckWare® has provided online cloud database storage. This app-based surgical e-logbook system was implemented and presented for trainees and graduates of CapaCare in November 2020. Training in the use of the app was limited and mostly performed through electronic platforms at the time of implementation. This was due to the COVID-19 pandemic, which limited the possibility of in-person training.

### The surgical e-logbook system

**Fig. 1:**
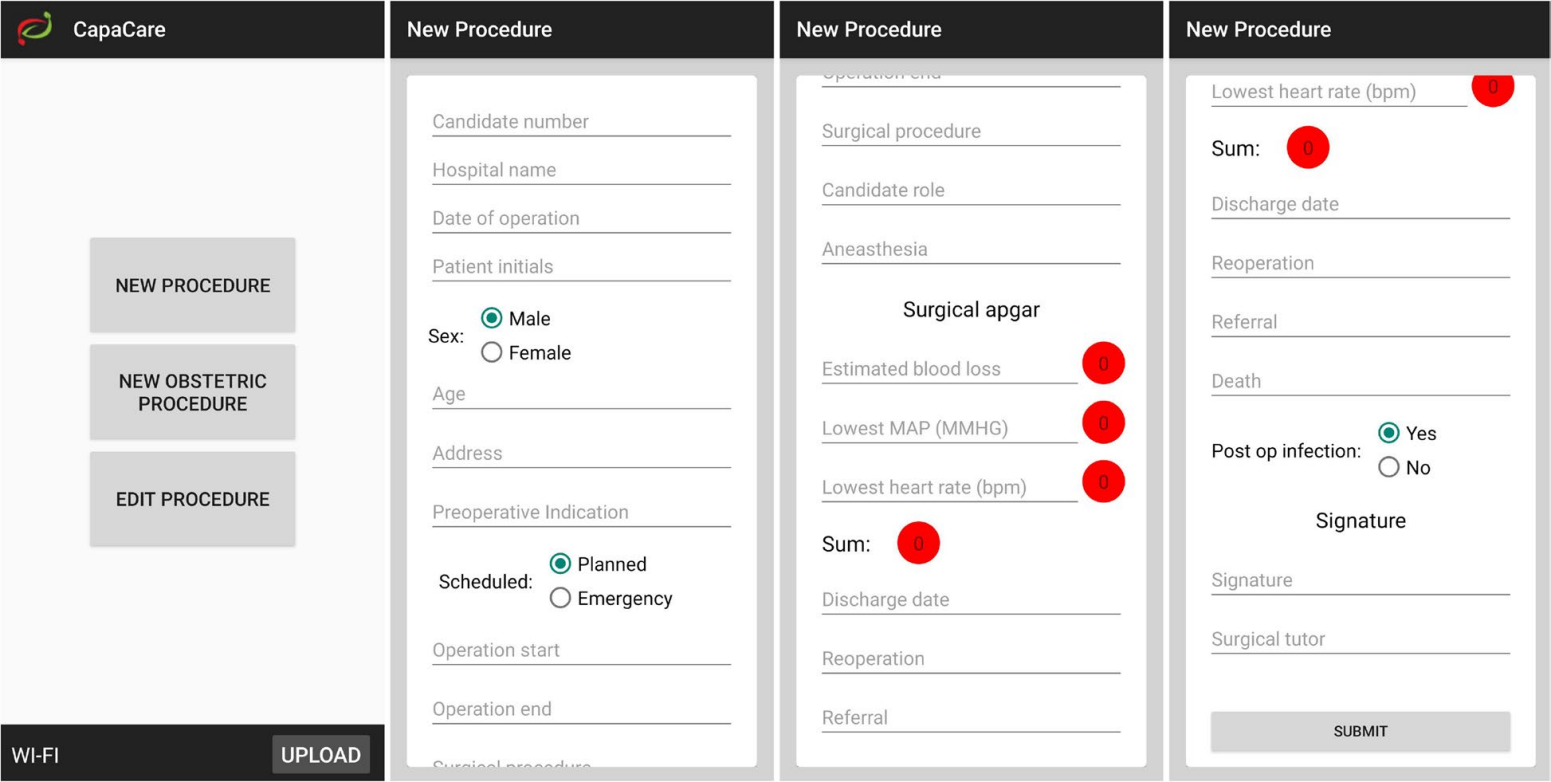
Screenshots of the surgical logbook application version 1.0

The users of the e-logbook system (trainees and graduates of the STP) register surgical procedures in the app after they have been performed. Information on the registrant, hospital, date of operation, patient sex and age, type of procedure, outcomes, and discharge date is entered in the e-logbook. The app’s user interface is presented in Fig. 1. Surgeries are entered in the surgical section of the app, while deliveries are entered in the obstetric section. In each section, information on the procedure is entered using predefined options in drop-down menus. These design choices are intended to limit errors that may occur if the information is entered as free text and to facilitate systematization and analysis of the data at both the user and hospital levels. Data entered by users in the app are automatically uploaded to the database after 6 weeks. This built-in delay allows users to make changes to the data and add information on outcomes and complications. Users may also upload their data to the database before this delay. As a privacy and security measure, users cannot access or make changes to procedures after they have been transferred to the e-logbook database.

### Selection and data collection

Study participants included all trainees and graduates of CapaCare who had uploaded at least one procedure to the e-logbook database during the observation period. The observation period varied by individual and was based on the date of each participant’s first and last entry in the app. Only procedures performed before February 27, 2021 and registered in the surgical section of the app were included. The study took place between November 2020 and February 2021.

The principal investigators, two fifth-year medical students from the Norwegian University of Science and Technology, were responsible for data collection. The HR data were collected with the help of local health workers employed at the study hospitals and have been previously described [16]. HRs were collected from all available hospitals where procedures had been registered in the e-logbook system. Depending on the routines for record-keeping at the study hospitals, the HRs could consist of various handwritten surgical and anesthetic operation theater books.

All participants using the e-logbook system were invited to take part in a semi-structured interview (Additional file 1) to gain insight into their use of the app, their experience with the app compared to the analog system, and to identify potential areas of improvement for the surgical e-logbook system. Interviews were conducted via telephone using Skype Credit in Microsoft® Skype™ to avoid unexpected financial costs for the participants. All interviews were conducted in English. Interviews were audio recorded and transcribed to facilitate the extraction of information.

### Data analysis

For the analysis of the quantitative data, the HRs were defined as the standard of reference. All available HRs were examined for procedures in each participant’s name. Only procedures registered at the hospital where the participant was posted during the individual observation period were included. The entries in the e-logbook database were cross-checked against entries in the HRs using predefined criteria for matching (Table 1) and categorized as a match, as overreported or as underreported. Information on the handling of specific cases can be accessed in Additional file 2.

**Table 1:** Criteria and categories for matching e-logbook entries with hospital records

Criteria:	Categories:
1. Name of the study participant recorded in the hospital record entry 2. Identical date of operation (+/− 1 day) 3. Identical patient ID^a^: a. Patient sex b. Patient age 4. Identical procedure	• Match: An entry was considered a match if the first and at least two of the remaining criteria were met • Overreported: E-logbook database entries that were not categorized as a match were considered overreported • Underreported: Procedures in the name of the participants in the HRs not meeting the criteria for a match were considered underreported

^a^Some study hospitals did not record patient sex in the hospital records (HRs). During the matching of these procedures, patient sex was deduced from the type of procedure performed, the given name of the patient, or both. Some study hospitals did not record patient age in the HRs. During the matching of these procedures, the only criterion used for matching patient IDs was “Patient sex”

All registered procedures were labeled as minor, major, or unspecified according to a predefined list (Additional file 3). Additionally, the procedures were labeled with student, intern (students and interns are collectively referred to as trainees), or graduate according to the participants’ progress in the STP.

The principal investigators categorized and analyzed the extracted information from the transcribed interviews according to the main themes.

## Results

### Quantitative analysis

**Fig. 2:**
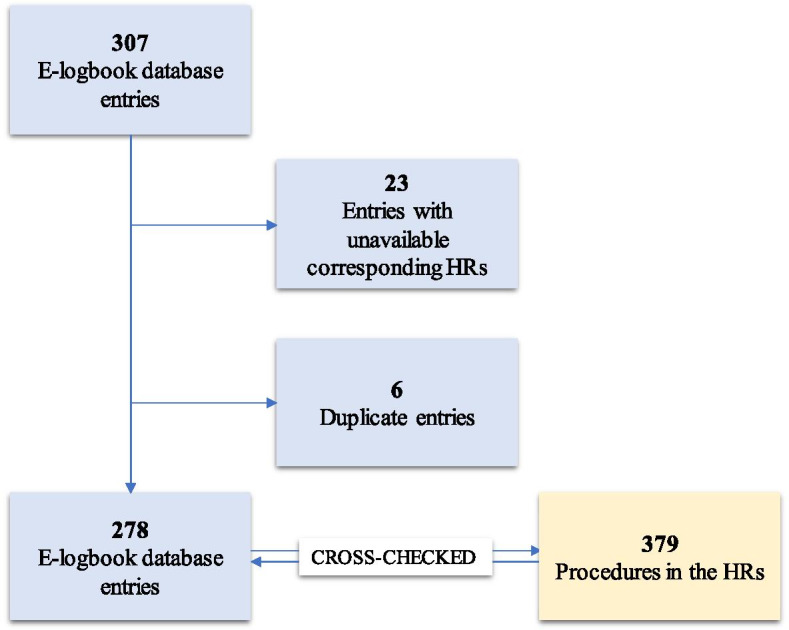
Cross-checking of e-logbook database entries and HR procedures

A total of 307 procedures were registered in the e-logbook database during the study period (Fig. 2). Of these, 23 were excluded because HRs were unavailable. An additional 12 procedures containing the same information were considered duplicate entries, of which six were excluded. The remaining 278 e-logbook database entries were eligible for cross-checking against the HRs. These entries consisted of data registered by a total of 14 participants representing nine hospitals. The number of recorded procedures for each individual ranged from two to 46. Five of the participants were students, four were interns, and five were graduates. Additionally, a review of the HRs from the study hospitals resulted in a total of 379 procedures registered in the names of the study participants.

After cross-checking, the primary analysis was performed based on the total number of included e-logbook database entries. This resulted in 67.3% matches and 32.7% overreported procedures. Additionally, a separate analysis of the HRs revealed that an estimated 50.7% of the procedures were underreported (Table 2).

**Table 2:** Results from cross-checking the e-logbook database against the HRs

E-logbook database				Hospital records	
		Match %	Overreporting %		Underreporting %
**Total**	***n*** **= 278**	**67.3**	**32.7**	***n*** **= 379**	**50.7**
**Procedure extent**^**a**^					
Major	*n* = 243	73.7	26.3	*n* = 361	50.4
Minor	*n* = 34	20.6	79.4	*n* = 16	62.5
**Participant progress**					
Grad	*n* = 81	76.5	23.5	*n* = 74	16.2
Intern	*n* = 109	75.2	24.8	*n* = 111	26.1
Student	*n* = 88	48.9	51.1	*n* = 194	77.8

^a^Unspecified procedures were excluded from the datasets during this analysis

*Grad* Graduate

In the study, minor procedures were found to be more likely to be overreported and less likely to be a match than major procedures. In addition, students were more likely to have underreported cases than both interns and graduates.

**Fig. 3:**
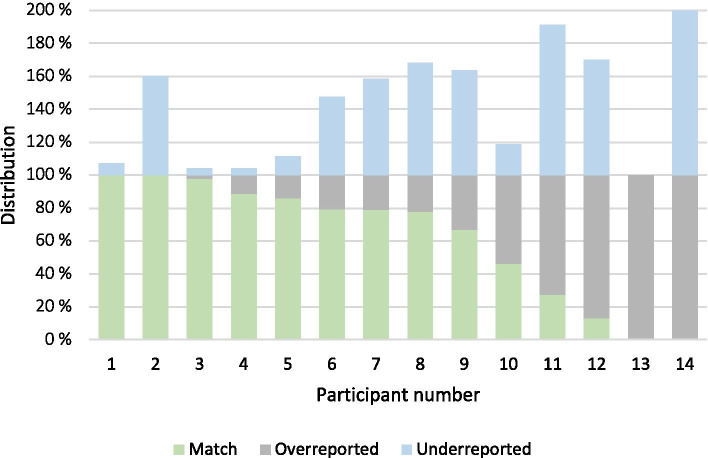
Distribution of matches, overreported, and underreported procedures for each participant

The observed distribution of individual match percentages ranged from 0 to 100% (Fig. 3). Similar variability can be seen in the proportion of underreported procedures from the HRs among the individual participants.

### Semi-structured interviews

Semi-structured interviews were performed with 12 of the 14 participants. Two were unable to participate due to personal reasons. The extracted information from the interviews is presented in Additional file 1. The direct quotations from the interviews presented in this section were slightly edited for readability.

All interviewed participants expressed overall satisfaction with the app, and all but one thought the app was easier to use than the analog logbook system. Several pointed out the advantage of avoiding the previous multistep process to hand in their logbook. All participants stated that the app was less time-consuming, which was favorable given the workload of some of the study hospitals. For instance, one participant stated, “This [app] is less time-consuming and that is very good for our program. Because we are very busy.” Several participants also valued the accessibility of the app:

Because we always have our phones, even at the theater our phone is close by us. You finish your surgery, [and] the first thing you do is to [pick up] your phone and start checking on what missed calls you have… And then you can think of, “Oh, I have the app,” and just fill in the information… It’s better than with the old system… So to me, going digital is really key at this time.

The app’s ease and accessibility were reflected by the reported average time interval between a procedure and registration in the app. Half of the participants registered procedures in the app immediately after surgery.

Although their experience with the app was generally positive, the participants had encountered several constraints. Limited experience with the technology presented a challenge: “I have not been used to using those apps with my phone ever before, this app is one of the first. So, it was a new thing to me, really.” Ten participants also had challenges with access to the internet. On the other hand, two participants expressed that the app presented a solution to this challenge: “With the app, you can [enter] your data, even without the internet.” Several found it difficult to download the app, which was not available on Google Play™ and had to be accessed from a third-party app provider at the time of the data collection: “I think that the access to the installation was the problem to me, but I think that was due to my technical know-how.” Participants also mentioned that better training for using the app could be useful.

The functional design of the app was also identified as a potential area of improvement by several participants. Six had encountered challenges related to the restricted options of indications and types of procedures included in the drop-down menus. As a result, some participants said they had submitted an entry with an incorrect indication or type of procedure. One participant stated, “I’m suggesting that we make expansions, at least for us to [be able to enter types of] procedures that are not in the app. For you to get the real picture of our [surgical activity].” On the other hand, the drop-down menus also presented an advantage, as they limited the amount of free text required to register a procedure: “[The app] includes less writing, then it includes less mistakes.” Additionally, the division of the app into a surgical and an obstetric section caused confusion among participants. This is exemplified by cesarean sections, which arguably could belong to both categories. Another concern was that the recorded procedures became inaccessible in the app after they had been uploaded to the database:

I am still thinking, one day the app will just get lost into my phone. So, … I am still recording my procedures in the [handwritten log-] book, that way it will not get lost there… Because the information I keep there is safer.

In addition to the technical challenges, including problems related to the app design, the registration of procedures was influenced by external factors. An example of this is the routine for the registration of procedures. During the interviews, some of the participants reported that someone else was assigned the task of recording the procedures in the HRs. Consequently, participants would not know if some surgeries they had attended were missing from the HRs.

A tendency was found for some participants to underreport minor procedures: “Smaller operations like chest drains and others, most of the time we don’t register them.” The same tendency was noted during an evaluation of the previously used analog system [14]. However, some also mentioned that this would change with the app, as it presents an easier way to register procedures: “Now that the app is available … I think a lot of [the minor procedures] will be captured.”

## Discussion

The evaluation of the app-based surgical e-logbook system, which found 67.3% matches with HRs and 32.7% overreported procedures, indicates a need to critically reflect on both the accuracy and the quality of this newly implemented digital system. Compliance with surgical logbook-keeping, be it analog or digital, in an environment with limited resources and technological know-how should also be critically examined. There might be several explanations for the discrepancies between the participants’ expressed satisfaction with the app and the low rate of accuracy found by the quantitative analysis. First, the e-logbook system was newly introduced and still at an early stage of adoption when data for this study were collected. The users were still in the process of getting to know the app and how to use it. However, this also opens up the possibility of learning from the most crucial time of implementation. Second, participants’ limited experience with app technology may have further decreased the percentage of matches. Some participants expressed that the information and training provided before they started to use the app were insufficient, and more training might increase the accuracy of registered procedures. Third, challenges related to the app design, particularly finding the right procedure in the drop-down menus, may have affected the process of cross-checking, as “identical procedure” was one of the study criteria for matching. Drop-down menus also pose a risk that users may mistakenly select the wrong option without noticing, causing errors in registration.

The heterogeneity among the participants’ distribution of match percentages indicates considerable individual variability in registration practices. In the case of two participants, 100% of the procedures were either overreported or underreported. For one of them, this is explained by a very limited number (*n* = 2) of procedures registered in the e-logbook database. For the other, it was explained during the interviews that procedures might not have been recorded in the HRs during a free hernia surgery camp, as someone else was assigned this task. All procedures of the participant in question were performed within the time span of this camp, leading to a potentially large number of overreported procedures. The distribution of underreported procedures also shows heterogeneity, and the percentage of underreported procedures (50.7%) is evidence that the app does not capture all procedures registered in the HRs. Reporting habits for cesarean sections is a possible reason for this, as these procedures might be registered in the obstetric section instead of the surgical section of the app, with only the latter being included in this study. A closer investigation revealed that some participants had, indeed, registered cesarean sections in the obstetric section. Cesarean sections constitute a large number of the entries in the HRs, as they are the second most frequently performed operation in the STP [17], which further emphasizes the importance of this issue.

A previous study that analyzed the analog logbook system within the same training scheme [14] found an estimated proportion of 73.1% (95% CI, 56–85) matching and close-matching entries; these categories combined are equivalent to the category “match” in the present study. A direct comparison between the studies suggests that the app-based system, with a 67.3% match rate, might be less accurate. A possible reason for the perceived lower accuracy of the e-logbook system, as mentioned above, is that the study was conducted shortly after its implementation. Moreover, the previous study from 2016 [14] is not necessarily an accurate representation of how the analog logbook system works today. At the time of the prior study, there were fewer trainees and graduates in the STP, and following up on the multistep analog system was easier. Furthermore, the methodology for the quantitative analysis was designed for the evaluation of the analog logbook system. To be able to compare the two systems, the methodology for cross-checking e-logbook entries with hospital records was maintained for the evaluation of the e-logbook system. However, it is not necessarily the most suitable study design for the quantitative analysis of the e-logbook system, as it may be more descriptive of the registration habits of the users than of the system itself. It might be argued, therefore, that the current results are not sufficient evidence to discard the e-logbook system.

During the participant interviews, it was revealed that some hospitals did not register minor procedures, as they are often performed on the wards and not in the operation theaters. Participants indicated that the app would capture more minor procedures, which would cause these procedures to be overreported in the cross-checking and, consequently, not be considered a match. However, this is not necessarily a weakness of the e-logbook system, as it will capture information that is missing from the HRs. The tendency of overreporting of minor procedures is further suggested by the subanalysis of the quantitative data.

The increased use of e-logbooks in LICs requires both technical competence and availability of technology, which have been identified as important barriers to the implementation of eHealth innovations [8]. Study participants described both of these barriers during the semi-structured interviews, and addressing these challenges might be crucial for a successful implementation. Although there are challenges in implementing an eHealth solution in a low-resource setting, this does not justify inaction. Digitalization is considered to be a driving force to improve access to surgical care and reduce health care inequality globally [8, 18]. It has the potential to increase workforce capacity, as technology automates work that was previously required to be performed by people [19]. A transition from a paper-based system to a digital system may also facilitate and increase the efficiency of patient care, especially when the amount of patient information is extensive. The substantial constraints on accessible health care in LICs, including the limited access to human resources, further justify the need to try innovative approaches.

### Strengths and limitations

Evaluating the e-logbook system at an early stage can be seen as a strength of the study. The insight gained from the assessment of eHealth solutions still under development can contribute to promoting user-friendliness and secure data management, leading to a successful implementation of the innovation [8]. In addition, the combination of qualitative and quantitative data enabled a fuller picture to be formed based on the available data. This approach is also supported by a Cochrane review that stated that more qualitative research is required to determine if and why eHealth solutions are successful [20].

A limitation of this study is the scarce amount of available quantitative data due to the short time between the implementation of the e-logbook system and the study period, resulting in a limited number of study participants, study hospitals, and procedures in the e-logbook database. Given the limited amount of data, detailed statistical analysis will add little value to the interpretation of the results. There is much to learn from descriptive statistics alone, which has therefore been presented instead. The use of different statistical methods complicates the comparison of the outcomes of this study to the evaluation of the analog logbook system. In addition, the reliability of the quantitative results as a representation of how the e-logbook system will work in the long term is questionable. Furthermore, the HRs collected for this study consisted exclusively of handwritten paper books. It has been shown that data collection from paper-based medical records in LICs varies in quality from inadequate [21, 22] to very accurate [23]. The assessment of the HRs was intricate, as the handwriting could be difficult to interpret, the information they contained varied between the study hospitals, and paper documents may have tears and creases that complicate the process. This may further have disrupted the cross-checking of the procedures, as the HRs are not a perfect standard of reference. This issue as well as the aforementioned constraints related to the sample size makes it difficult to draw conclusions based on the quantitative analysis.

However, an equally representative group of trainees and graduates of the STP were included during the evaluation of both the analog and the e-logbook systems, which contributes to the value of this study. This was the case for both the quantitative analysis and the semi-structured interviews, which is an advantage in regard to the comparison of the systems. Nevertheless, the interviews for the evaluation of the e-logbook system were conducted via telephone. The lack of nonverbal communication, such as gestures and facial expressions, may have caused important cues to go unnoticed. For both evaluative studies, cultural differences and linguistic barriers may have had a similar effect on communication.

### Key recommendations

Based on the results and discussion of this article, the following key recommendations can be made:

- A proper introduction, sufficient training, and mentoring on the use of the e-logbook system should be offered, including guidance on the use of the necessary technology and the app itself.
- Access to the necessary technology, including devices compatible with the app, reliable internet access, and power to charge devices should be ensured.
- Further development of the app design and modifications to achieve greater user-friendliness. A potential alteration is the inclusion of a free-text field where the user can add additional information if necessary, for example as a supplement to the drop-down menus.
- Enabling users to easily retrieve their own procedures registered in the e-logbook system. This information should be anonymized to adhere to data protection regulations and confidentiality.
- The distinction between the surgical and obstetric sections of the e-logbook system should be clarified for users, and information on which type of procedures should be entered into each section should be provided.

## Conclusions

A reliable documentation system is necessary to evaluate and monitor the quality of health workforce training in low-resource settings. An app-based surgical e-logbook system was developed to assist in this task and to substitute for a previous analog system. Early evaluation of this e-logbook system provided crucial information to facilitate guidance and adjustment of further implementation. The data from the quantitative analysis show that the accuracy of the e-logbook system should be improved. However, accuracy might increase with usage over time. Interviews with users of the new fully digital system indicated that it was a much-needed innovation but also identified important areas for improvement, including the need for more training in the use of the system, modifications to improve user-friendliness, and better access to the necessary technology.

## Supplementary Information

**Additional file 1.** Semi-structured interview. Extracted information from the semi-structured interviews conducted with study participants.

**Additional file 2.** Handling of specific cases. Overview of how some specific cases were handled while cross-checking e-logbook database entries with the HRs.

**Additional file 3.** Minor and major procedures. Predefined list used for categorizing procedures as minor, major, and unspecified during the data analysis.

## Data Availability

The data collected for this study contain confidential information and cannot be made public. A summary of the interview responses can be accessed in Additional file 1. The data are available on request from the corresponding author, SS.

## Abbreviations

LIC: Low-income country
HR: Hospital record
STP: Surgical Training Programme
COVID-19: Coronavirus Disease 2019
CI: Confidence interval
