# Supplementary material for: Early evaluation of the transition from an analog to an electronic surgical logbook system in Sierra Leone
Source: BMC Med Educ. 2021 Nov 15;21:578. doi: 10.1186/s12909-021-03012-z (PMC8591157; doi:10.1186/s12909-021-03012-z)
Supplement: Supplementary file 1 — Additional file 1. Semi-structured interview. Extracted information from the semi-structured interviews conducted with study participants. [file 12909_2021_3012_MOESM1_ESM.docx]

Additional file 1

Semi-structured interview

Semi-structured interviews were performed with 12 of the 14 participants.

| Feedback on the app | |
| --- | --- |
| 1. Have you encountered any problems related to the app? | |
| - 5 reported having problems with downloading the app | |
| - 6 reported having problems related to the automatic upload after six weeks, with the entries no longer being accessible in the app as a consequence | |
| - 2 of them reported delaying the submission of procedures to avoid the procedures being uploaded before complete information on the case was available | |
| - 10 reported having problems with entering information | |
| - 6 of them were unable to find the correct indication or type of procedure in the drop-down menus | |
| 2. Have you encountered any problems concerning IT equipment or internet access while using the app? | |
| - 10 reported lack of internet access being a problem | |
| - 1 reported having been unable to charge the device used with the app due to lack of electricity | |
| - 2 answered “no” | |
| Comparison to the analog logbook system | |
| 3. What do you think about the app compared to the analog logbook system? | |
| - All 12 reported being overall satisfied with the app | |
| - 6 stated that the app is more accessible | |
| - 5 found it advantageous that they avoided the previous multistep process to hand in their logbook | |
| 4. Do you think the app has made it easier or more difficult to register procedures? | |
| - The app has made it easier | 11/12 |
| - The app has made it more difficult | 0/12 |
| - Ambiguous response | 1/12 |
| 5. Do you think you use more or less time with the app compared to the analog logbook system? | |
| - Use more time with the app | 0/12 |
| - Use less time with the app | 12/12 |
| 6. Since you started using the app, have you exclusively used the app, or have you gone back to using the analog logbook system? | |
| - Have exclusively used the app | 9/12 |
| - Have used both the app and the analog logbook system | 3/12 |
| - Have gone back to the analog logbook system | 0/12 |
| 7. What do you think about the user manual and the information you got before you started to use the app? | |
| - It was clear and/or useful | 6/12 |
| - Was not able to access the manual | 3/12 |
| - Was able to access the manual but did not use it or found it difficult to follow | 3/12 |
| Registration of procedures | |
| 8. A surgical procedure can be categorized as either major or minor. Is there a difference as to how you register these procedures? | |
| - Yes, a tendency to underreport the minor procedures | 3/12 |
| - Yes, a previous tendency to underreport the minor procedures, but this has changed with the app | 2/12 |
| - No | 5/12 |
| - Ambiguous response | 2/12 |
| 9. After you started using the app, have you attended any procedures that have not been registered in the app? | |
| - Yes | 2/12 |
| - No, but have registered some procedures with a wrong indication or type of procedure due to limitations in the app (see question 1) | 3/12 |
| - No | 7/12 |
| 10. Have you registered any procedures in the app that have not been registered in the hospital records? | |
| - Yes | 2/12 |
| - Maybe; someone else is assigned the task of recording the procedures in the hospital records | 2/12 |
| - No | 8/12 |
| 11. Have you attended any procedures that have been registered neither in the app nor in the hospital records? | |
| - Yes | 3/12 |
| - 1 of them said this was in relation to pilot surgeries, 1 said it could occur due to workload and 1 said some minor procedures could be missed | |
| - No | 9/12 |
| 12. Have you registered any procedures, either in the app or in the hospital records, that have not taken place? | |
| - Yes | 0/12 |
| - No | 12/12 |
| 13. What is the average time interval between the date of a procedure and registration in the app? | |
| - Immediately | 6/12 |
| - Less than 1 day | 1/12 |
| - 1-6 days | 3/12 |
| - 7-20 days | 1/12 |
| - 21-90 days | 0/12 |
| - More than 90 days | 0/12 |
| - Don't know | 1/12 |
