# Supplementary material for: Early evaluation of the transition from an analog to an electronic surgical logbook system in Sierra Leone
Source: BMC Med Educ. 2021 Nov 15;21:578. doi: 10.1186/s12909-021-03012-z (PMC8591157; doi:10.1186/s12909-021-03012-z)
Supplement: Supplementary file 2 — Additional file 2. Handling of specific cases. Overview of how some specific cases were handled while cross-checking e-logbook database entries with the HRs. [file 12909_2021_3012_MOESM2_ESM.docx]

Additional file 2

Handling of specific cases

The HRs from one hospital were unavailable for the study. 23 entries in the app were identified to have been performed at this hospital. The 23 entries in question were therefore deemed non-eligible for cross-checking. To avoid misclassifying these entries, the entries were excluded from the study.

One of the participants entered procedures using the wrong participant ID. This was identified before the data was analyzed, and the e-logbook database entries were updated with the correct participant ID.

Two entries in the e-logbook database did not contain any essential information. These were considered incomplete database entries and were disregarded for the analysis. Consequently, these are not included in Figure 1.

The HRs from one hospital were missing operation dates for a period, during which one participant had their individual start date. The participant in question had one procedure registered in their name in the HRs during this period, and it was consequently not possible to know whether this procedure took place during the participant’s individual observation period. To avoid missing any potential underreporting, the procedure was included for the data analysis.
