# Supplementary material for: Early evaluation of the transition from an analog to an electronic surgical logbook system in Sierra Leone
Source: BMC Med Educ. 2021 Nov 15;21:578. doi: 10.1186/s12909-021-03012-z (PMC8591157; doi:10.1186/s12909-021-03012-z)
Supplement: Supplementary file 3 — Additional file 3. Minor and major procedures. Predefined list used for categorizing procedures as minor, major, and unspecified during the data analysis. [file 12909_2021_3012_MOESM3_ESM.docx]

Additional file 3

Minor and major procedures

| **Major procedures:**  Above-knee amputation  Appendectomy  Below-ankle amputation  Below-knee amputation  Caesarean section  Colostomy  Craniotomy  Cystectomy  Dental extraction  Evacuation of pyoartitis  External fixation  Femoral hernia repair  Finger amputation  Foreign body removal  Hand amputation  Hysterectomy  Inguinal hernia repair  Internal fixation  Laparotomy  Large bowel resection  Myomectomy  Orchidectomy  Osteomyelitis debridement  Perforated ulcer operation  Repair of bladder injury  Repair of ruptured uterus  Repair of uterine tear  Salpingectomy for ectopic pregnancy  Scrotal hydrocele  Skin-graft  Small bowel resection  Strangulated hernia repair  Toe amputation  Tubal ligation  Umbilical hernia repair  Uterus prolapse operation  Ventral hernia repair  Vesicovaginal fistula repair | **Minor procedures:**  Breech extraction  Chest tube  Cleaning and dressing  Dental other  Dilatation and curettage  Evacuation of hematoma  Excision of lipoma  Excision other  Incision and drainage  Induction of labour  Instrumental delivery (vacuum extraction)  Male circumcision  Manual placenta removal  Manual vacuum aspiration  Necrotectomy/wound debridement  POP casts for fractures  Removal of corpus alienum  Repair of cervical/vaginal/perineal tears  Reposition of joint  Secondary closure  Skeletal traction  Skin traction  Suprapubic puncture  Urethral catheterization  Urethral dilation for urethral stricture  Uterus tamponade for postpartum  haemorrhage  Wound suturing |
| --- | --- |
|  | **Unspecified:**  Neurosurgery other  Obstetrics/gynaecology other  Orthopaedic surgery other  Soft tissue surgery other  Unknown |
